# Effects of neural aging on speech perception in bilinguals vs. monolinguals

**DOI:** 10.3389/fnhum.2026.1913294

**Published:** 2026-08-05

**Authors:** Miwako Hisagi, Beatriz Barragan, Margaret Winter, Annett Acosta, Burgundy Bisson, Alicia Park, Melody Arakelian, Susan Lee, Alessandro Presacco

**Affiliations:** 1 Department of Communication Disorders, Doctor of Audiology Program, California State University-Los Angeles, Los Angeles, CA, United States; 2 Department of Speech-Language Pathology, AT Still University, Mesa, AZ, United States; 3 Department of Otolaryngology, University of Southern California, Los Angeles, CA, United States

**Keywords:** aging, bilingualism, mismatch negativity, speech perception, vowel discrimination

## Abstract

This study examined the effects of aging and bilingualism on neural speech perception in Spanish-English (SE) bilinguals and American English (AE) monolinguals. As the population of bilingual older adults continues to grow, understanding how bilingual experience influences auditory processing across the lifespan has important theoretical and clinical implications. Electrophysiological measures, including mismatch negativity (MMN) and the sensory obligatory cortical responses (P1–N1–P2 complex), were used to assess early auditory encoding and automatic speech discrimination during passive perception of unfamiliar Japanese vowel contrasts. Participants included younger and older AE monolinguals and SE bilinguals. All participants completed a passive listening EEG task, followed by a phonological memory task, and a second EEG session using the same speech stimuli. Analyses focused on MMN and P1–N1–P2 amplitude, latency, and scalp distribution. Overall, older AE monolinguals demonstrated delayed MMN responses in the left hemisphere and midline compared to both younger monolinguals and bilinguals. In contrast, older SE bilinguals exhibited a delayed MMN response only at a single frontal site when compared to younger bilinguals. While age-related decline in auditory processing was evident, particularly regarding MMN latency, older bilinguals demonstrated relatively preserved MMN responses compared to monolinguals peers. The findings suggest a dissociation between early sensory encoding and automatic speech discrimination processes. Older bilingual adults demonstrated enlarged N1 amplitudes relative to all other groups, potentially reflecting age-related sensory changes, while simultaneously maintaining relatively preserved MMN responses. These findings suggest that lifelong bilingualism may support more adaptable auditory discrimination mechanisms during aging, potentially contributing to greater neural resilience or cognitive reserve. This study contributes to a growing body of evidence suggesting that bilingualism may shape auditory and cognitive processing across the lifespan. The findings also highlight the importance of considering bilingual experience in the assessment and rehabilitation of older adults, particularly in increasingly diverse clinical populations.

## 1 Introduction

The global population is aging rapidly, and the proportion of bilingual older adults, particularly Spanish-English (SE) speakers in the United States, is steadily increasing. This demographic shift presents new challenges and opportunities for understanding how aging influences cognitive and auditory processes. Although bilingualism and aging have each been studied extensively, there is limited research on how they jointly affect the neural mechanisms underlying auditory speech processing, particularly when comparing SE bilinguals and American English (AE) monolinguals across age groups.

Each language has a unique phonological system that shapes the range of speech sounds its speakers can readily distinguish. While first-language (L1) speech perception is typically automatic and robust to attentional demands, second-language (L2) perception requires more effort (e.g., Datta et al., 2020; Hisagi et al., 2015). For SE bilinguals whose L1 is Spanish, lexical decisions often reflect L1 phonological constraints across languages (Freeman et al., 2021), whereas AE monolinguals process speech without such cross-linguistic interference. Hisagi et al. (2024) demonstrated that early sequential SE bilinguals shift between L1 and L2-based processing strategies depending on task difficulty. In a nonword length discrimination task, SE bilinguals relied on English rules for easier trials but reverted to Spanish rules under challenging conditions. These findings suggest that bilingual speech processing strategies are dynamic and influenced by both language experience and task demands. Successful comprehension in a L2 depends on the ability to perceive novel, nonnative speech sounds (Intartaglia et al., 2016; Kissling, 2015). However, the innate capacity to distinguish nonnative phonemic contrasts declines sharply after the first year of life (Kuhl, 2010; Mattys et al., 2005; Sebastián-Gallés and Soto-Faraco, 1999). Beyond this critical period, recognizing phonemic contrasts relies on slower, more resource-demanding cognitive mechanisms, which in turn hinders efficient L2 word recognition (Burgaleta et al., 2014).

While language processing studies provide valuable insights in younger bilingual populations, fewer have examined how aging modifies these processes. The likelihood of hearing loss increases with age and is closely linked to a decline in cognitive processes and speech comprehension (Wingfield et al., 2005). In addition to these potential auditory challenges, bilingual individuals face processing demands linked to reduced exposure to, or experience with, their L2 (Cowan et al., 2022). As a result, older bilingual adults may experience greater difficulty with word recognition. For example, Hisagi et al. (2024) found that older SE bilinguals performed poorly compared to monolingual peers on vowel discrimination tasks, suggesting that reliance on native-language acoustic cues may interfere with speech perception in aging bilingual listeners.

Educational level appears to be an important factor influencing auditory processing, particularly through its relationship with cognitive functions. Years of schooling and academic achievement have been shown to contribute to auditory attention and memory abilities (Agrawal et al., 2025). For instance, individuals with higher levels of education demonstrate better performance on dichotic sentence identification tasks (de Andrade et al., 2015). In the context of aging, Murphy et al. (2016) reported that among adults aged 50 to 87, higher educational attainment was positively associated with performance on auditory processing measures. Importantly, they suggested that this relationship is more likely driven by the cognitive demands of the tasks rather than by differences in auditory sensory function per se. Consistent with this interpretation, Moreno-Gómez et al. (2017) found that in older adults with presbycusis, higher educational level, particularly when combined with musical training, was associated with reduced deficits in music discrimination. Together, these findings highlight the role of education as a cognitive reserve factor that may mitigate age-related changes in auditory processing.

### 1.1 Neurophysiological measures: mismatch negativity (MMN) in speech perception

Previous research on L2 speech perception rely mainly on behavioral measures, which, although informative, are limited in their ability to directly index the underlying brain processes that distinguish L1 from L2 perception, or reveal subtle differences across groups (Morrison and Taler, 2020). In contrast, neurophysiological methods such as event-related potentials (ERPs) provide millisecond-level temporal resolution, enabling the examination of auditory and cognitive processes as they unfold before their behavioral manifestation (Bellis et al., 2000; García et al., 2018; Rugg and Coles, 1995).

Of particular relevance is the mismatch negativity (MMN), an ERP component that reflects the brain’s automatic detection of deviations in an auditory sequence (Näätänen et al., 2007; Näätänen et al., 2014). MMN typically occurs 150–200 ms post-stimulus and is widely interpreted as an index of pre-attentive auditory discrimination. Importantly, MMN can be recorded while attention is directed away from the auditory modality, making it an excellent measure of automaticity in speech perception (Hisagi et al., 2010). Larger MMN amplitudes and earlier latencies are generally associated with more efficient and automatic auditory processing, whereas smaller amplitudes and prolonged latencies indicate weaker or less automatic neural representations. The MMN is modulated by the ease with which a sound change is detected: more salient changes typically elicit larger amplitudes and shorter latencies. Previous studies have reported smaller MMNs to segmental and duration contrasts in non-native compared with native listeners during passive listening tasks in which participants ignored the auditory stimuli while watching a muted video or reading a book (Dehaene-Lambertz et al., 2000; Menning et al., 2002; Nenonen et al., 2003; Nenonen et al., 2005; Ylinen et al., 2006). Because the MMN provides an objective measure of pre-attentive auditory discrimination and is highly sensitive to experience-dependent neural plasticity, it has been widely used to assess changes resulting from language exposure and targeted auditory training (Näätänen et al., 2014).

Research has demonstrated that bilinguals often show smaller and delayed MMN responses compared to monolinguals (García and Froud, 2018), and that aging further reduces MMN amplitudes while prolonging latencies (Näätänen et al., 2014). Iino et al. (2018) reported that speech stimuli elicited larger amplitudes and shorter latencies than pure-tone stimuli, suggesting differential processing of linguistically relevant information at early stages of auditory cortical processing. Their findings also demonstrated age-related declines in MMN responses for pure-tone stimuli, whereas similar age effects were not observed for vowel-speech stimuli, indicating that long-term exposure to speech sounds may modulate pre-attentive neural processing differently than non-speech sounds. Similarly, studies using pure tones have shown consistent age-related reductions in MMN amplitude, although latency effects have been less consistent (Schiff et al., 2008). Taken together, previous MMN findings suggest that aging may affect automatic speech and auditory processing differently, and that the extent of decline is influenced by the characteristics and linguistic relevance of the stimuli used.

### 1.2 Neurophysiological measures: P1–N1–P2 cortical auditory evoked response potential (CAEP)

The P1–N1–P2 complex of the cortical auditory evoked potential (CAEP) reflects early cortical encoding of acoustic stimuli and provides a well-established index of age-related changes in central auditory processing. Although the P1-N1-P2 complex is characterized by delayed latencies and larger responses in older adults (Tremblay et al., 2003) suggesting degraded auditory temporal processing at the cortical level (Alain et al., 2014; Sörös et al., 2009; Lister et al., 2016), this pattern is not uniform and appears to depend on the characteristics of the stimuli. A study by Tremblay et al. (2004) reported prolonged N1 latencies in older adults when processing complex or rapidly changing stimuli, whereas this age-related difference was absent when simpler stimuli, such as tones or speech presented at slower rates, were used. This suggests that refractory properties of the auditory system may differ with age, particularly under conditions of increased processing demands. P2 latencies remained longer in older adults for speech stimuli regardless of presentation rate, while no such differences with young adults emerge for tonal stimuli (Tremblay et al., 2004). Together, the findings indicate that age-related changes in the P1–N1–P2 complex are modulated by stimulus complexity and temporal demands.

Amplitude findings, however, are less uniform and appear to depend on stimulus type, inter-stimulus interval, task demands, and the degree to which peripheral hearing status is controlled. Several investigations report increased amplitudes of early components (P1 and N1) in older adults, potentially reflecting reduced inhibitory control or compensatory cortical hyperexcitability (Ng et al., 2022; Haumann et al., 2023; Alain and Snyder, 2008). McCullagh and Shinn (2013) found increased N1 and P2 amplitudes in older adults compared to younger adults in quiet or favorable SNR conditions, but no significant differences were found between the groups at more difficult SNRs. In contrast, later components such as P2 were found to be reduced in amplitude and delayed in older adults, suggesting diminished efficiency in later-stage auditory processing (Wang et al., 2024; Oliveira et al., 2019). Importantly, interpretation of age-related CAEP differences requires careful consideration of peripheral hearing status. When audibility is equated, some latency and amplitude differences are attenuated, indicating that both peripheral and central factors contribute to observed effects (McClannahan et al., 2019).

Collectively, these findings indicate that aging is associated with altered cortical responsiveness, characterized in some paradigms by enhanced early sensory activity alongside reduced efficiency at later stages of processing. This pattern is consistent with models proposing diminished inhibitory regulation and compensatory neural recruitment within the aging auditory cortex (de Villers-Sidani et al., 2010). The P1–N1–P2 complex provides a measure of obligatory early cortical encoding of acoustic input, reflecting the integrity and timing of sensory processing, whereas the mismatch negativity (MMN) indexes automatic detection of phonetic change and the integrity of auditory discrimination mechanisms. Together, these complementary ERP measures allow researchers to examine both the fidelity of early cortical encoding and the automaticity of speech contrast processing.

Research in young bilingual adults (Hajimohammadi and Heidari, 2025) has shown shorter CAEP latencies, particularly in the P1 and N1 components, compared to monolinguals, with no significant group differences in amplitude. These latency reductions have been attributed to experience-dependent neuroplasticity associated with managing two language systems, leading to enhanced neural synchrony and more efficient transmission and processing of auditory input.

By leveraging CAEP and MMN measures in tandem, researchers can assess not only whether discrimination of L2 contrasts occur, but also whether neural processing approaches the automaticity characteristic of L1 representations. This combined neurophysiological framework offers a more sensitive and mechanistically grounded approach than behavioral measures alone, enabling a more precise characterization of auditory aging and bilingual speech perception across the lifespan.

### 1.3 Present study

Despite substantial evidence that bilingualism, aging, and cognitive load each shape auditory speech processing, their combined effects remain insufficiently understood. The present study addresses this gap by examining how bilingual experience and age jointly influence automatic speech perception. Younger and older AE monolinguals and SE bilinguals were compared using electrophysiological measures, including cortical auditory evoked potential (CAEP) and mismatch negativity (MMN) recorded during a passive vowel discrimination task. To minimize lexical influences, the task employed unfamiliar Japanese vowel contrasts. Japanese nonsense words (/tado/ and /taado/) were selected because they differ only in vowel duration, allowing temporal speech cues to be manipulated while minimizing lexical and semantic influences.

Specifically, the study was guided by three primary research questions. First, do AE monolinguals and SE bilinguals differ in MMN and CAEP indices of speech processing? Second, how does aging affect MMN and CAEP responses, and do these age-related changes differ between monolinguals and bilinguals? Third, does prior cognitive load, induced through a phonological memory Japanese nonword repetition (NWR) task (Gupta, 2003), modulate MMN responses, reflecting potential fatigue or changes in neural engagement, and do these effects vary as a function of age and language group?

For MMN, younger adults were expected to show larger amplitudes and shorter latencies than older adults. AE monolinguals were also expected to demonstrate larger and earlier MMN responses than SE bilinguals, reflecting stronger automatic phonological representations. Thus, older SE bilinguals were expected to exhibit the weakest MMN responses overall. For the P1–N1–P2 complex, older adults were expected to show prolonged latencies and larger amplitudes relative to younger adults, reflecting age-related changes in auditory cortical processing. Because CAEP primarily reflects early sensory encoding, language-group differences were expected to be relatively small. To examine the effects of cognitive demand, MMN responses were compared across two sessions separated by a phonological memory task. Cognitive load or fatigue was expected to reduce MMN amplitudes, particularly in older adults, although increased phonological engagement could alternatively enhance MMN responses.

## 2 Methods

### 2.1 Participants

The study included 26 younger adults (18–39 years) and 23 older adults (50 + years) who were either American English monolinguals (Mono) or Spanish-English bilinguals (Bi). Participants were assigned to four groups: Group 1(YMono)—Younger monolinguals with normal hearing (*n* = 14); Group 2 (YBi)—Younger bilinguals with normal hearing who acquired Spanish as their native language and learned English later in childhood (*n* = 12); Group 3 (OMono)—Older monolinguals with normal to mild sensorineural hearing loss (SNHL; *n* = 11); and Group 4 (OBi)—Older bilinguals with normal to mild SNHL who acquired Spanish as their native language and learned English later (*n* = 12). Although the sample size was relatively small (*n* = 11 ~ 14 per group), it is comparable to those reported in previous studies of speech perception and auditory processing. For example, Hisagi et al. (2025) included two groups of 15 participants each, Shafer et al. (2021) included three groups of 12, 12, and 11 participants, and both Hisagi et al. (2010) and Hisagi et al. (2015) included two groups of 12 participants. Therefore, the sample size used in the present study is consistent with those commonly employed in this area of research.

Mono participants had minimal exposure to any foreign language and spoke only English in their daily lives. Notably, mild SNHL does not interfere with MMN responses (Bruckmann et al., 2021). A study by Oates et al. (2002) showed that sensorineural hearing loss primarily affects higher-level cortical (“nonsensory”) processing, whereas earlier sensory responses, such as the MMN, are comparatively preserved. Although mild hearing loss may result in modest prolongation of MMN latency, substantial reductions in MMN amplitude are typically observed only with greater degrees of hearing loss. The mean three-frequency pure-tone average (3PTA: 500, 1 k, 2 k Hz) was 6.29 dB HL for YMono, 7.59 dB HL for YBi, 18.64 dB HL for OMono, and 20.00 dB HL for OBi. SNHL was present in 6 of 11 OMono participants and 10 of 12 OBi participants.

All participants were recruited on a voluntary basis via self-selection sampling from the Southern California region. Screening procedures included negative histories for neurological pathologies and otorhinolaryngological surgeries, as well as normal otoscopic examination findings. Participants with any history of neurological disease or psychiatric disorder were excluded from the study. Table 1 presents participant information for each group.

**Table 1:** Participant’s demographic and language background information.

	Younger		Older	
	MONO (*N* = 14)	Bi (*N* = 12)	MONO (*N* = 11)	Bi (*N* = 12)
Age mean	28	28.92	64.45	61.83
Gender	6M8F	3M9F	4M7F	2M10F
Mean (four linguistic domains)	9.939	9.333	10	9.476
Age of English Acquisition	0.72	4.55	0.5	4.43
Age of English fluency	2.82	7.33	2.55	5.86

YMono, Young monolingual group; YBi, Young bilingual group; OMono, Older monolingual group; OBi, Older bilingual group; SD, standard deviation.

Language background and proficiency were self-reported through an online Qualtrics survey modeled after those used in Hisagi et al. (2025). The survey included questions on perceived English proficiency across four domains: reading, writing, speaking, and speech understanding, using a 0–10 Likert scale, where 0 represented no proficiency and 10 indicated “native-like” proficiency. Overall, bilinguals rated their English proficiency lower than monolinguals.

### 2.2 Instruments and measures

#### 2.2.1 The Montreal cognitive assessment (MoCA)

Cognitive function was measured using Version 7.1 of the Montreal Cognitive Assessment (MoCA; Nasreddine et al., 2005), administered in either English or Spanish according to participant’s preference; three participants completed the assessment in Spanish, and the remainder forty-six completed it in English. Based on the official MoCA 7.1 framework, the test covers seven cognitive domains (“Montreal Cognitive Assessment (MoCA): Concept and clinical review,” 2013): Visuospatial/Executive (trail making, cube copying, clock drawing), Naming (identification of three low-familiarity animals), Attention (digit span forward and backward, vigilance, serial 7 subtraction), Language (sentence repetition, verbal fluency), Abstraction (similarity comparisons), Delayed Recall (recall of five previously presented words without cues), and Orientation (date, month, year, day, place, city). Total scores range from 0 to 30, with higher scores reflecting better cognitive performance. In accordance with MoCA scoring guidelines, participants with 12 years or fewer of formal education were granted one additional point to adjust for educational differences. The MoCA was included as a descriptive measure of participants’ cognitive status and was not used as a screening or eligibility criterion. Both groups fell within the normal cognitive screening range, with only a small difference observed between the mean MoCA scores of the older bilingual (26.40) and older monolingual (27.00) groups. Table 2 presents average MoCA scores for each group.

**Table 2:** MoCA average scores by group.

	Younger		Older	
	MONO (*N* = 14)	Bi (*N* = 12)	MONO (*N* = 11)	Bi (*N* = 12)
MoCA mean	28.21	25.70	27.00	26.40

#### 2.2.2 Stimuli

The target stimuli were nonsense words differing in vowel duration, produced by a native Japanese female speaker with the Tokyo dialect. The standard is /aa/ as in /taado/ and the deviant is /a/ as in /tado/. These stimuli have been used successfully in previous studies and provide a well-controlled means of examining temporal cue processing. Because neither English nor Spanish uses phonemic vowel length in the same way as Japanese, the stimuli reduce the influence of participants’ native-language lexical knowledge and permit evaluation of temporal cue perception across language groups. Frequent standard stimuli were presented 85% of trials, while deviant stimuli occurred 15%. To direct attention away from the auditory stimuli, a visual oddball paradigm was presented simultaneously, in which participants viewed images of a happy dog or cat (standard) and a crying dog or cat (deviant). The auditory stimuli and experimental paradigm followed Hisagi et al. (2010).

#### 2.2.3 Nonword repetition (NWR) task

A stimulus recorded by a native Japanese female speaker, with a signal-to-noise ratio of at least 10 dB, was used. The stimulus consisted of two sequences of 3 (e.g., genugi), 5 (e.g., toginudage), 7 (e.g., tadekenukidago), and 9 syllables (e.g., tadekonutonugiteda). Participants were asked to repeat Japanese nonwords of increasing syllable length, designed to maintain cognitive engagement and assess the effects of fatigue and increased cognitive load. The Nonword Repetition task was included solely as a cognitive load manipulation and was not intended as a behavioral outcome measure. Therefore, repetition accuracy was not analyzed in the present study.

#### 2.2.4 EEG

For the EEG preparation, the participant’s head was measured to select the appropriate cap size. A 32-electrode research-grade EEG amplifier cap (V-amp, Brain Products GmBH) was fitted, with hair parted to ensure gel to contact with the scalp. Impedance was measured and reduced 20 *Ω* before the participant was seated in a sound booth. During the task, participants were instructed to ignore the auditory stimuli while identifying the sad cats and dogs in the visual display.

### 2.3 Procedure

The procedure followed a systematic sequence. 1) Participants provided written informed consent after reviewing a form describing the study procedure and expectation, in accordance with the Declaration of Helsinki and with approval from the California State University, Los Angeles Institutional Review Board; 2) Participant completed a language background questionnaire; 3) Otoscopic examination was conducted to rule out outer ear abnormalities; 4) Pure-tone audiometry was administered to verify hearing status, and the resulting pure-tone averages were used to confirm participant eligibility (normal hearing to mild sensorineural hearing loss); 5) The Montreal Cognitive Assessment (MoCA) was conducted; 6) Initial EEG trial consisting of 7 blocks presenting standard and deviant stimuli; 7) Participant’s performance on the phonological nonword repetition task; 8) Second EEG trial consisting of 7 blocks presenting standard and deviant stimuli. The complete experimental session duration was approximately 2 h.

E-Prime (version 3.0) running on a desktop PC was used to control auditory stimulus presentation and deliver event markers to the EEG acquisition computer for time-locking EEG activity to speech sound onsets. Auditory stimuli were presented at 75 dB SPL in sound field through two speakers positioned 110 cm from the participant’s head at 50-degree angles to the left and right. The distractor visual oddball paradigm was presented on a laptop PC using E-Prime (version 3.0). The laptop was placed on the table, with the top of the screen positioned approximately 50 cm from the participant’s eyes at a 15-degree downward angle.

### 2.4 Data analysis

The EEG data were collected and analyzed via BrainVision Analyzer, at a 250 Hz sampling rate. MMN of at least 0.5 microvolts in amplitude was needed to be considered as a mismatch. Although there were 32 channels, the focus was placed on the three frontal and three central sites because these primary channels produce the most robust MMN. MMN responses (with amplitudes of at least 0.5 μV) typically appeared in a fronto-central distribution, peaking around 200 ms, within a window of 150–250 ms. post-deviant onset (Näätänen et al., 2007).

Raw EEG data were first referenced to Tp9. In the few cases where the Tp9 channel was excluded because of excessive noise, Tp10 was used as the reference instead. The data were then band-pass filtered between 1 and 30 Hz using a fourth-order, zero-phase Butterworth filter. Any noisy channels were visually inspected and removed if present. Eye-movement artifacts were addressed using Independent Component Analysis (ICA) with the Infomax algorithm (Makeig et al., 2004).

A time window between 150–250 ms was selected for MMN analyses. For averaging, 500 artifact-free sweeps were used for standard stimuli and 75 for deviant (target) stimuli. Artifact rejection was set at ±50 μV and, if necessary, was gradually increased by a ± 5 μV step until the desired number of sweeps was reached. The analysis window spanned from −56 ms to 944 ms. MMN was computed by subtracting the averaged ERP waveform for standard stimuli from that of the deviant stimuli. These measures were analyzed across six fronto-central electrode sites: left hemisphere (F3, C3), midline (Fz, Cz), and right hemisphere (F4, C4).

The following time windows were selected for P1-N1-P2 analysis: P1 (35–80 ms) N1 (80–150 ms) and P2 (150–250 ms). Standard stimuli were used for this analysis, as this response is often elicited by it. Between-group differences in the latency and amplitude of the MMN and P1–N1–P2 complex were examined using linear mixed-effects models (LMMs) conducted in IBM SPSS Statistics. The primary objective of these analyses was to evaluate differences across groups while accounting for sources of within-subject variability and potential confounding factors.

For the mismatch negativity (MMN), separate LMMs were specified for latency and amplitude. In each model, participant was included as a random effect to account for repeated measurements across electrode sites. Fixed effects included Group (Ymono, YBi, OMono, and OBi) and electrode location (F3, Fz, F4, C3, Cz, and C4), allowing for the assessment of whether group-related differences varied across scalp regions. Education was included as a covariate to control for its potential influence on auditory processing, given the variability in educational attainment across participants.

The same analytic approach was applied to the P1–N1–P2 complex. Separate models were conducted for latency and amplitude for each component (P1, N1, and P2), maintaining the same structure of fixed and random effects and inclusion of education as a covariate. Significant effects involving Group were further explored using Benjamini-Hochberg (BH) procedure *post hoc* comparisons to allow for sensitive detection of theoretically motivated group differences while maintaining consistency with the planned comparison framework of the study.

## 3 Results

The analyses focused on identifying group differences among younger monolinguals (YMono), younger bilinguals (YBi), older monolinguals (OMono), and older bilinguals (OBi) in the latency and amplitude of the MMN and P1–N1–P2 complex across six electrode sites, F3, Fz, F4, C3, Cz, and C4. To account for potential confounding effects, education level was included as a covariate using a linear mixed-effects modeling (LMM) approach. One participant from the OBi group was excluded due to extreme N1 amplitude values, approximately four times larger than the group mean across multiple electrode sites, exceeding typical variability and indicating a non-representative outlier. Importantly, analyses conducted both with and without this participant yielded the same pattern of results, confirming that the exclusion did not influence the overall findings.

### 3.1 Cognitive load

Two EEG sessions were conducted for each participant: one prior to and one following a cognitive fatigue–inducing task (Japanese non-word repetition). Visual inspection of the data indicated highly similar latency and amplitude values across the two sessions. To formally evaluate potential cognitive load or fatigue effects, paired-samples *t*-tests were conducted comparing latency and amplitude for all ERP components (MMN, P1, N1, and P2) across the six electrode locations. These analyses revealed no significant differences (*p* > 0.05) between the pre and post-task sessions for any component or measure.

Given the absence of session-related effects, the two observations per participant were averaged to obtain a single, more stable estimate of latency and amplitude for each ERP component at each electrode site. These averaged values were subsequently used as the dependent variables in the following linear mixed-effects models.

### 3.2 MMN

#### 3.2.1 MMN latency

**Figure 1:**
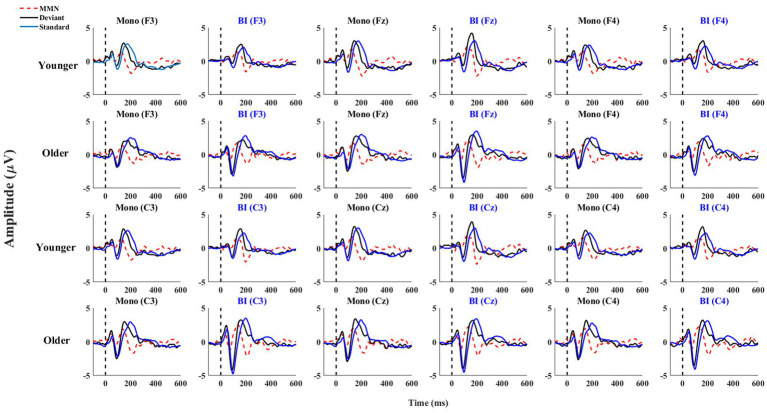
MMN waveforms for younger and older monolinguals and bilinguals in frontal and central electrode locations. Event-related potentials evoked by a standard (blue) nonword sound and a deviant (black) nonword sound. The difference waveform (dotted) delineates the Mismatch Negativity (MMN) component, identified in younger and older monolinguals (Mono) and bilinguals (BI) in six electrode sites, frontal left (F3), frontal midline (Fz), and frontal right (F4), central left (C3), central midline (Cz), and central right (C4).

When analyzing the MMN latency (see Table 3 for descriptive data), the LMM revealed a significant Group × Location interaction, *F*(15, 1,675) = 13.22, *p* < 0.001, ηp^2^ = 0.106, indicating that differences in MMN latency between groups varied as a function of electrode site. Follow-up comparisons showed that group differences were not uniform across the scalp but instead emerged at specific locations. In particular, the OMono group exhibited delayed MMN latencies relative to YMono group at F3 (*p* = 0.02), Fz (*p = 0*.04), and Cz (*p* = 0.005), and relative to YBi at F3 (*p* = 0.002), Fz (*p = 0*.04), C3 (*p* = 0.005), Cz (*p* = 0.001), and C4 (*p* = 0.029). The OBi group exhibited delayed MMN latencies only relative to YBi in F3 (*p = 0*.037). No differences between the young groups or the older groups were found. Overall, these results indicate group differences in MMN latency reflecting delayed responses in OMono adults in the left hemisphere and midline, in both frontal and central sites, in contrast, only one difference involving OBi was found in comparison to the younger groups (Figure 1).

**Table 3:** MMN latency and amplitude descriptive data.

	Location	YMono		YBi		OMono		OBi	
		Mean	SD	Mean	SD	Mean	SD	Mean	SD
Latency (second)	F3	0.213	0.014	0.205	0.008	0.221	0.017	0.209	0.017
	Fz	0.213	0.015	0.211	0.012	0.219	0.010	0.210	0.016
	F4	0.212	0.018	0.212	0.010	0.215	0.017	0.206	0.022
	C3	0.212	0.014	0.202	0.007	0.219	0.014	0.211	0.012
	Cz	0.208	0.016	0.202	0.009	0.216	0.013	0.207	0.011
	C4	0.215	0.021	0.208	0.012	0.217	0.014	0.211	0.011
	Total	0.212	0.017	0.207	0.011	0.218	0.014	0.209	0.015
Amplitude (µV)	F3	−2.522	1.020	−2.083	0.626	−2.092	0.999	−2.242	1.162
	Fz	−2.952	1.143	−2.896	0.965	−2.447	1.154	−2.884	1.627
	F4	−2.586	1.155	−2.289	0.737	−2.129	0.999	−2.512	1.437
	C3	−2.455	1.031	−2.378	0.881	−2.436	1.533	−2.783	1.028
	Cz	−2.726	1.312	−3.007	0.980	−2.636	1.383	−2.685	1.305
	C4	−2.438	1.217	−2.560	0.961	−2.681	1.524	−2.685	1.379
	Total	−2.613	1.159	−2.535	0.924	−2.404	1.297	−2.632	1.345

YMono, oung monolingual group; YBi, Young bilingual group; OMono, Older monolingual group; OBi, Older bilingual group; SD, standard deviation.

The absence of a significant Group × Education interaction, *F*(3, 41.08) = 1.43, *p* = 0.247, suggests that education did not systematically moderate group differences in MMN latency across participants. The significant three-way interaction Group × Location × Education, *F*(15, 1675.00) = 12.22, *p* < 0.001, ηp^2^ = 0.099, likely reflects variability in the association between education and latency across electrode sites rather than a theoretically meaningful modification of group effects.

#### 3.2.2 MMN amplitude

The LMM for MMN amplitude revealed a significant Group × Location interaction, *F*(15, 1,675) = 5.91, *p* < 0.001, ηp^2^ = 0.050. In line with the analytical approach, interpretation focused on the interaction to determine whether amplitude differences between groups varied across electrode sites. However, follow-up *post hoc* comparisons did not reveal significant differences between groups at any electrode location (see Table 3 for descriptive data). Instead, the observed interaction was driven by differences across locations within each group rather than by systematic differences between groups. These findings do not provide evidence for meaningful group-related variation in MMN amplitude, rather, shows spatial distribution patterns that differ within groups, without translating into contrasts across groups (Figure 1).

Exploratory examination of within-group patterns revealed distinct interesting topographical trends. At frontal sites, both younger groups exhibited larger MMN amplitudes over the left hemisphere compared to the right (YMono *p* = 0.015; YBi *p* = 0.006). In contrast, older monolinguals showed no significant hemispheric differences (*p* > 0.05), whereas older bilinguals demonstrated a significant asymmetry in the opposite direction, with larger amplitudes over the right hemisphere relative to the left (OBi *p* = 0.031). A broadly similar pattern was observed at central sites. Larger amplitudes over the left hemisphere were evident in younger bilinguals (*p* = 0.009) and older monolinguals (*p* = 0.008), and the same directional trend was present, though not statistically significant, in younger monolinguals (*p* > 0.05). In contrast, older bilinguals again showed the reverse pattern, with significantly greater amplitudes over the right hemisphere compared to the left (*p* = 0.001).

Overall, although these within-group spatial patterns are noteworthy, the absence of significant between-group differences indicates that MMN amplitude does not robustly differentiate the young and old monolingual and bilingual groups.

### 3.3 P1-N1-P2 complex

#### 3.3.1 P1 latency and amplitude

The LMM for the P1 latency revealed a significant Group × Location interaction, *F*(15, 1,675) = 10.56, *p* < 0.001, ηp^2^ = 0.086. The follow-up *post hoc* comparisons did not reveal significant differences between groups at any electrode location. Instead, the observed interaction was driven by differences across locations within each group, providing evidence for no group-related variation in P1 latency.

Exploratory examination of within-group patterns revealed an interesting topographical trend at frontal sites. The younger monolingual group showed earlier responses on the left hemisphere compared to the right (*p* = 0.028). In contrast, young and old bilinguals exhibited earlier response on the right hemisphere relative to the left (Y-Bi *p* = 0.001; O-Bi *p* = 0.013), and the same directional trend was present, though not statistically significant, in older monolinguals (*p* > 0.05).

**Figure 2:**
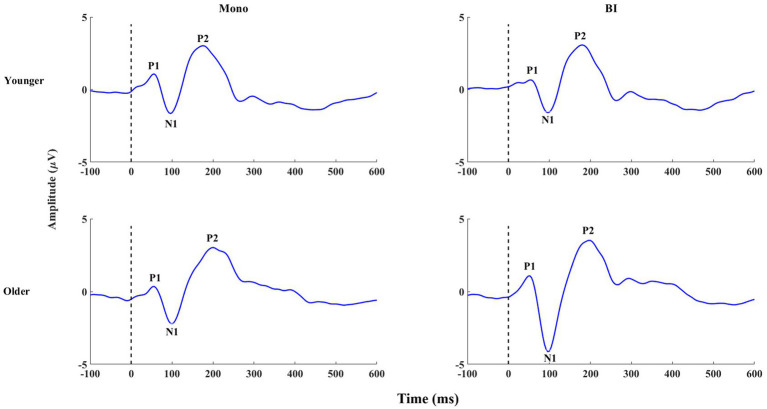
Group averaged cortical auditory evoked potential (CAEP) waveforms illustrating P1, N1, and P2 components. Waveforms elicited from younger and older monolinguals (Mono) and bilinguals (BI) by nonword stimuli.

For the P1 amplitude, the LMM revealed a significant Group × Location interaction, *F*(15, 1,675) = 27.77, *p* < 0.001, ηp^2^ = 0.199. Follow-up comparisons showed larger P1 amplitudes for older bilinguals compared to older monolinguals in F3 (*p* = 0.037) and C3 (*p* = 0.024.). No other significant differences between groups were found (Figure 2).

#### 3.3.2 N1 latency and amplitude

The LMM for N1 latency revealed a significant Group × Location interaction, *F*(15, 1,675) = 4.11, *p* < 0.001, ηp^2^ = 0.036. The follow-up *post hoc* comparisons did not reveal significant differences between groups at any electrode location. Instead, the observed interaction was driven by differences across locations within each group, providing evidence for no group-related variation in N1 latency. The examination of within-group patterns did not provide evidence for meaningful trends.

When analyzing N1 amplitude, the LMM revealed a significant Group × Location interaction, *F*(15, 1,675) = 41.60, *p* < 0.001, ηp^2^ = 0.271, indicating differences between groups varying as a function of electrode site. Follow-up comparisons showed the OMono group exhibited larger N1 amplitude relative to younger monolinguals at Fz (*p = 0*.026), C3 (*p* = 0.031), Cz (*p* = 0.005), and C4 (*p* = 0.016). OBi group showed significantly larger N1 amplitudes relative to all other groups in the frontal locations (F3 YMono *p* < 0.001; F3 YBi *p* < 0.001; F3 OMono *p* = 0.022; Fz YMono *p* < 0.001; Fz YBi *p* < 0.001; Fz OMono *p* = 0.004; F4 YMono *p* < 0.001; F4 YBi *p* < 0.001; F4 OMono *p* = 0.011), and significantly larger N1 amplitudes relative to younger groups in the central locations (C3 YMono *p* < 0.001; C3 YBi *p* = 0.004; Cz YMono *p* = 0.007; C4 YMono *p* = 0.002; C4 YBi *p* = 0.009). No differences between the young groups were found. Overall, these results indicate group differences in N1 amplitude reflecting larger responses in older adults, especially in older bilinguals in both frontal and central sites (Figure 2).

#### 3.3.3 P2 latency and amplitude

The LMM for P2 latency revealed a significant Group × Location interaction, *F*(15, 1,675) = 7.86, *p* < 0.001, ηp^2^ = 0.066. The follow-up *post hoc* comparisons showed the older monolingual group exhibited delayed P2 latencies compare to the young monolingual (*p = 0*.009) and young bilingual (*p = 0*.016) groups in the F3 location, and delayed P2 latency compared to the young bilingual group (*p = 0*.039) in Fz. No other significant differences between-groups were found.

For the P2 amplitude, the LMM revealed a significant Group × Location interaction, *F*(15, 1,675) = 15.02, *p* < 0.001, ηp^2^ = 0.113. Follow-up post hoc comparisons did not reveal significant differences between groups at any electrode location. Instead, the observed interaction was driven by differences across locations within each group without any specific trends.

Overall, the analyses of the P1–N1–P2 complex revealed limited evidence of robust between-group differences across components. The most notable effect emerged for N1 amplitude, where older adults, particularly the older bilingual group, exhibited larger amplitudes compared to younger groups. This finding represents the primary source of group-related variation within the complex (Figure 2).

No other consistent or meaningful between-group differences were observed for P1 or P2 components, either in latency or amplitude. Although several significant interactions were identified in the models, these effects were primarily driven by differences across electrode locations within each group. As such, they reflect variations in spatial distribution patterns rather than true contrasts between groups. The results indicate that group differences within the P1–N1–P2 complex are selective and component-specific, with the clearest effect observed in N1 amplitude (Figure 2), while other effects largely reflect topographical variability that does not translate into systematic between-group differences.

## 4 Discussion

The present study investigated the perception of temporally cued vowel contrasts in younger and older monolingual and bilingual adults to better understand how aging and bilingualism interact to shape automatic speech perception. Specifically, the study examined neural responses associated with early auditory encoding and automatic speech discrimination through the analysis of mismatch negativity (MMN) and the cortical auditory evoked potential (CAEP) P1–N1–P2 complex. Overall, the findings revealed important interactions between age and linguistic background, suggesting that bilingualism may influence how the aging auditory system processes speech sounds at both sensory and discrimination levels.

For the MMN, older monolingual adults demonstrated significantly delayed latencies compared to both younger groups, while older bilingual adults differed significantly only from younger bilinguals. These findings are consistent with previous literature showing that aging is associated with prolonged MMN latencies, reflecting slower auditory discrimination, reduced neural processing speed, and a general decline in central auditory processing efficiency (Cheng et al., 2013). The present results replicate the expected age-related effects described in previous studies (Iino et al., 2018; Näätänen et al., 2014), as both older groups exhibited slower responses than their younger linguistic counterparts. However, an important pattern emerged when comparing monolingual and bilingual participants. Bilinguals demonstrated a MMN latency trend, in all electrode locations, shorter than monolinguals. Most notably, the magnitude of the age-related delay appeared smaller in the older bilingual group than in the older monolingual group.

This pattern suggests that bilingualism may confer a protective or enhancing effect on auditory neural responsiveness during aging. Such interpretation aligns with previous findings indicating that bilingual individuals exhibit enhanced neural encoding and more efficient attentional control during speech processing (Krizman et al., 2012). Managing two language systems across the lifespan may strengthen executive control mechanisms and auditory attention processes, facilitating more efficient processing of speech stimuli. Astheimer et al. (2016) similarly reported that bilinguals engage attentional control mechanisms more efficiently than monolinguals during speech-processing tasks. From a broader cognitive perspective, the constant activation, inhibition, and switching between two languages may continuously recruit executive control networks, contributing to enhanced cognitive flexibility (Kroll and Bialystok, 2013). Additionally, bilingualism has been associated with increased cognitive reserve, which may allow older bilingual adults to maintain more efficient neural functioning despite age-related neurobiological decline (Bialystok et al., 2012).

Although no significant group differences were observed in MMN amplitude, the latency findings remain highly relevant. Earlier MMN latencies are generally interpreted as reflecting more rapid and automatic auditory discrimination processes (Näätänen et al., 2007). Therefore, the relatively preserved MMN timing observed in bilingual adults suggests faster and potentially more efficient neural processing of speech sounds. Contrary to our initial hypothesis that older monolinguals would exhibit earlier and stronger MMN responses due to more robust automatic phonological representations, the findings revealed a different pattern. Specifically, the shorter MMN latencies observed in older bilinguals suggest that bilingualism may confer a distinct neural processing advantage during the early stages of auditory perception, potentially counteracting age-related slowing within auditory processing mechanisms.

An additional important finding was the spatial distribution of the MMN effects. The primary group differences were observed in the left hemisphere and midline electrode sites, regions commonly associated with linguistic and phonological processing. This pattern suggests that participants were not simply responding to acoustic differences between sounds, but rather processing the stimuli as linguistically meaningful speech contrasts despite the fact that the stimuli were nonsense Japanese words. The left-lateralized effects reinforce the interpretation that bilingual experience influences higher-order speech processing mechanisms rather than only basic auditory detection.

The analyses of the P1–N1–P2 complex provided complementary information regarding age-related changes in central auditory processing. Overall, limited between-group differences were found for P1 and P2 components. The most notable effect emerged for N1 amplitude, where older adults, particularly older bilinguals, showed significantly larger amplitudes compared to younger participants. Specifically, the N1 amplitude of the older monolingual group was significantly larger than that of younger monolinguals, while the older bilingual group exhibited significantly larger N1 amplitudes than all other groups, including older monolinguals.

Interpretation of N1 amplitude changes in aging has been inconsistent across the literature, likely due to methodological differences involving stimulus complexity, interstimulus interval, task demands, and peripheral hearing control. Nevertheless, several studies have reported enhanced early auditory component amplitudes in older adults, particularly for P1 and N1, suggesting reduced inhibitory control or compensatory cortical hyper excitability associated with aging (Ng et al., 2022; Haumann et al., 2023; Alain and Snyder, 2008). Similarly, McCullagh and Shinn (2013) observed increased N1 and P2 amplitudes in older adults under quiet or favorable signal-to-noise conditions, although these differences diminished under more demanding listening conditions.

The combined interpretation of the MMN and P1–N1–P2 complex findings is particularly informative. The P1–N1–P2 complex reflects obligatory early cortical encoding of acoustic input and provides insight into the integrity and timing of sensory auditory processing. In contrast, the MMN indexes automatic detection of phonetic change and the integrity of auditory discrimination mechanisms. The response pattern observed in older bilingual adults is therefore especially noteworthy. On one hand, the larger N1 amplitudes may reflect age-related changes in sensory processing integrity or compensatory cortical recruitment. On the other hand, the relatively preserved MMN responses suggest maintained automatic speech discrimination abilities. Together, these findings indicate that although aging may alter early sensory encoding processes, bilingual experience may support preservation of higher-order auditory discrimination mechanisms. This pattern supports the possibility that bilingualism contributes to resilience in auditory processing even in the presence of age-related neural decline. Future research comparing pure-tone stimuli, which are less linguistically relevant, and speech stimuli, which are linguistically meaningful, would help clarify how bilingualism influences pre-attentive auditory processing (e.g., Iino et al., 2018).

The phonological memory task was included as a cognitive load manipulation designed to examine whether increased cognitive demands would negatively affect auditory processing, particularly in older adults. It was expected that older participants would demonstrate diminished MMN responses following the task due to cognitive fatigue. Contrary to expectations, all groups remained relatively stable across EEG sessions. These results may indicate that the phonological memory task did not generate sufficient cognitive fatigue or that participants were not equally engaged with the task demands. It is important to clarify that because the phonological memory task was implemented strictly as a cognitive load manipulation rather than a performance metric, behavioral data were not collected. Our electrophysiological results demonstrated that this task failed to induce measurable cognitive fatigue, as EEG measures remained highly comparable between the pre and post-task blocks. These findings highlight the importance for future research to rigorously pilot such phonological memory tasks within the target population to ensure that the intended thresholds for cognitive fatigue are successfully reached. Nevertheless, cognitive load remains an important factor to consider in auditory aging research. Older adults without overt cognitive impairment may still exhibit slowed or altered auditory processing under cognitively demanding conditions (e.g., Pichora-Fuller et al., 1995; Peelle, 2018; Wingfield and Tun, 2007), and the extent to which bilingualism interacts with these effects remains unclear. Future studies incorporating more demanding cognitive manipulations may provide important insights into the interaction between bilingualism, cognitive fatigue, and auditory neural processing in aging populations.

An additional factor that should be considered when interpreting the present findings is the presence of mild sensorineural hearing loss (SNHL) in a substantial proportion of the older participants. Although several studies have reported little or no effect of mild SNHL (e.g., Bruckmann et al., 2021; Oates et al., 2002), age-related hearing loss has been associated with alterations in neural speech processing throughout the auditory pathway, including reduced temporal precision and changes in the representation of speech signals at both subcortical and cortical levels (Presacco et al., 2019). Therefore, some portion of the age-related differences observed in the present study may reflect the combined influence of aging and peripheral hearing loss.

However, previous work suggests that age-related neural processing deficits cannot be fully explained by peripheral hearing loss alone. Presacco et al. (2019) reported that older adults, regardless of hearing status, exhibited weaker neural representations of speech compared with younger adults, whereas hearing loss primarily influenced more subtle aspects of auditory system organization, including the relationship between midbrain and cortical processing. These findings suggest that central auditory aging and peripheral hearing loss may independently contribute to changes in neural speech processing. Consequently, although the presence of SNHL among older participants should be acknowledged as a potential contributing factor, the observed MMN latency differences are unlikely to be attributable solely to hearing sensitivity and may instead reflect broader age and experience-related differences in auditory neural processing.

An important methodological strength of the present study was the inclusion of education as a covariate in the analyses. Previous research has demonstrated that higher educational attainment is positively associated with auditory processing performance in older adults (Murphy et al., 2016). In the current sample, the older monolingual group included participants with graduate and doctoral-level education, whereas the older bilingual group included individuals with substantially lower educational backgrounds, including elementary-level education. Controlling for education was therefore critical to reduce potential confounding effects. Interestingly, significant Group × Location × Education interactions emerged in many analyses. The *post hoc* analyses suggested that these effects were largely driven by within-group spatial differences rather than direct between-group comparisons. Nonetheless, the findings point to a potentially important interaction between aging, bilingualism, and educational experience in shaping auditory neural processing; however, the notable disparities in educational attainment between the groups ultimately limit the interpretation of these results. This complex interaction warrants further investigation in future research.

Overall, the present findings contribute important evidence regarding the neural mechanisms underlying speech perception in aging bilingual populations. Additional research examining how cognitive and auditory processes are shaped by bilingualism in older adulthood is necessary to better understand non-pathological aging in multilingual populations. Investigating second-language speech perception provides valuable information about auditory neural processing mechanisms and contributes to the development of more accurate neurocognitive models of bilingual speech perception. Although the combined effects of aging and bilingualism on speech processing remain largely not understood, continuing to investigate these interactions is essential for improving auditory rehabilitation and reducing barriers to effective hearing healthcare in increasingly diverse aging populations.

### 4.1 Limitations and future directions

Several limitations should be considered when interpreting the present findings. First, the relatively small sample size within each group may limit the generalizability of the results. Although significant patterns emerged across behavioral and electrophysiological measures, larger and more diverse samples would improve statistical power and increase the applicability of the findings across broader bilingual and aging populations. In addition, while important bilingual variables such as self-reported language proficiency and age of acquisition were considered, other relevant within-group factors were not systematically examined. Variables including language dominance, frequency of language use, code-switching habits, and sociolinguistic context may substantially influence cognitive flexibility, auditory processing, and neural responsiveness, and therefore, should be incorporated in future research designs.

Another important limitation relates to the auditory stimuli used in the study. The use of a Japanese nonsense word was intentionally selected to ensure unfamiliarity across all participants and minimize lexical bias. However, this choice may also have introduced variability related to phonetic complexity, perceptual salience, or unfamiliar acoustic patterns that do not fully represent naturalistic speech processing. Although the use of controlled nonwords provides important experimental advantages, future studies would benefit from including a broader range of ecologically valid speech stimuli and more realistic listening conditions, including speech-in-noise paradigms that better reflect everyday communication challenges experienced by older adults.

The phonological memory task implemented for cognitive fatigue manipulation presented an important limitation for the study. While intended to elevate cognitive load and expose shifts in auditory neural processing, the lack of significant EEG changes across sessions indicate the task may not have been demanding enough to elicit measurable cognitive fatigue. Future research would benefit from incorporating more rigorous cognitive challenges combined with task performance metrics and objective or subjective fatigue indices to validate the efficacy of the manipulation. Such approaches may provide a more sensitive understanding of how cognitive effort and fatigue influence auditory processing in aging bilingual populations.

Another potential limitation is that the older bilingual group presented with a slightly lower mean MoCA score than the older monolingual group. However, this small difference is unlikely to have functional implications, given that the MoCA is a global cognitive screening tool rather than a precise measure of pre-attentive auditory processing. Future studies should further examine the interplay between global cognitive screening and neurophysiological indices of auditory processing in bilingual aging.

An additional limitation of the present study concerns the lack of educational consistency across our sample, particularly within the older adult groups where monolinguals were more highly educated than their bilingual counterparts. Although education was included as a covariate in our statistical analyses to mitigate this variance, previous research has firmly established that educational background significantly influences auditory processing in older cohorts (e.g., Agrawal et al., 2025. de Andrade et al., 2015, Murphy et al., 2016). Consequently, future studies should aim to recruit samples that are more evenly balanced in terms of educational attainment to ensure greater internal validity.

A further consideration is that the older bilingual group was, on average, approximately 3 years younger than the older monolingual group. Although both groups fell well within the predefined older adult age range, advancing age can influence auditory processing speed. Future research utilizing stricter age-matching groups or statistical adjustments for age, would isolate the effects of bilingualism from chronological age.

Future research should continue exploring the long-term effects of bilingualism on auditory aging to determine whether bilingual experience functions as a protective factor against age-related auditory and cognitive decline. Longitudinal studies combining behavioral, cognitive, and neurophysiological measures would be especially valuable for tracking changes in auditory processing across the lifespan and identifying potential mechanisms of neural resilience. Future studies should also compare performance using non-native temporal-contrast stimuli and native English and Spanish speech materials to determine whether bilingual advantages in temporal processing extend across different linguistic contexts. Additionally, future work should examine whether targeted cognitive or auditory training interventions can produce sustained improvements in auditory processing efficiency and speech perception in older adults. Understanding how bilingual experience interacts with neural plasticity and auditory rehabilitation may ultimately contribute to the development of more effective and culturally responsive clinical interventions for aging bilingual populations.

## 5 Conclusion

The present study contributes to a growing body of literature examining the interaction between aging and bilingualism in auditory speech processing. By combining electrophysiological measures of early sensory encoding and automatic speech discrimination, the findings provide evidence that bilingual experience influences neural processing across the lifespan. Although age-related slowing in auditory processing was observed, particularly in MMN latency, bilingual older adults demonstrated relatively preserved neural speech discrimination compared to monolingual older adults. These findings suggest that lifelong management of two languages may support more efficient or adaptable auditory processing mechanisms during aging.

Importantly, the results revealed a dissociation between sensory encoding and automatic discrimination processes. Older bilingual adults exhibited enlarged N1 amplitudes, potentially reflecting age-related changes in sensory processing, while simultaneously maintaining relatively preserved MMN responses. This pattern suggests that bilingualism may confer resilience in specific auditory discrimination mechanisms even in the presence of age-related neural decline. The findings therefore support a more nuanced understanding of cognitive and auditory aging, recognizing bilingualism not only as a linguistic experience but also as a factor capable of shaping neural adaptability and cognitive reserve.

The study also has important clinical implications. As the population of bilingual older adults continues to rapidly increase, understanding how bilingualism influences auditory perception becomes critical for developing accurate and equitable assessment and rehabilitation practices. The findings highlight the importance of considering bilingual experience when interpreting auditory processing measures in older adults. Overall, this work advances current knowledge of the neural mechanisms underlying speech perception in aging bilingual adults and underscores the importance of continuing to investigate how bilingual experience interacts with auditory and cognitive aging. Further research integrating behavioral, cognitive, and neurophysiological approaches will be essential for refining models of bilingual speech processing and improving clinical care for increasingly diverse aging populations.

## Data Availability

The raw data supporting the conclusions of this article will be made available by the authors, without undue reservation.
